# Molecular docking analysis of furfural and isoginkgetin with heme oxygenase I and PPARγ

**DOI:** 10.6026/97320630017356

**Published:** 2021-02-28

**Authors:** Preetha Santhakumar, Lavanya Prathap, Anitha Roy, Selvaraj Jayaraman, M Jeevitha

**Affiliations:** 1 Department of Physiology, Saveetha Dental College and Hospitals, Saveetha Institute of Medical and Technical Sciences, Chennai - 600 077, Tamil Nadu, India; 2 Department of Anatomy, Saveetha Dental College and Hospitals, Saveetha Institute of Medical and Technical Sciences, Chennai - 600 077, Tamil Nadu, India; 3 Department of Pharmacology, Saveetha Dental College and Hospitals, Saveetha Institute of Medical and Technical Sciences, Chennai - 600 077, Tamil Nadu, India; 4 Department of Biochemistry, Saveetha Dental College and Hospitals, Saveetha Institute of Medical and Technical Sciences, Chennai - 600 077, Tamil Nadu, India; 5 Department of Periodontics, Saveetha Dental College and Hospitals, Saveetha Institute of Medical and Technical Sciences, Chennai - 600 077, Tamil Nadu, India

**Keywords:** Furfural, isoginkgetin, anti-inflammation, molecular docking

## Abstract

It is of interest to document the molecular docking analysis based binding data of furfural and isoginkgetin with heme oxygenase I and PPARγ in the context of inflammation for further consideration in drug design and development.

## Background

When released from intracellular heme-containing proteins in excessive quantities, heme is potentially harmful [1]. Oxidative and inflammatory damage associated with the pathology of different disorders may be caused by the released heme, or free heme [2]. Therefore at sites of damage, it is most important to eliminate excess free heme. The first and rate-limiting step of the oxidative degradation of free heme to produce carbon monoxide (CO), ferrous iron (Fe2+), and biliverdin (BV) [3] is catalyzed by the microsomal enzyme heme oxygenase (HO). The BV formed in this reaction is then converted by a BV reductase into bilirubin (BR), and the ferrous iron is rapidly sequestered and recycled for heme synthesis by ferritin [4]. Two genetically different HO isozymes have been described to date: an inducible form, heme oxygenase-1 (HO-1), and a constitutively expressed form, heme oxygenase-2 (HO-2) [5]. HO-1, once expressed under different pathological conditions, has the capacity to metabolise large quantities of free heme in order to generate high concentrations of its enzymatic by-products and, as a result, are capable of affecting different biological events and have recently attracted substantial medical attention [6]. HO-1 can be expressed not only by its free heme substrate, but also by a wide spectrum of pro- inflammatory factors, indicating that HO-1 plays other essential roles in the resolution of inflammation, in addition to its central function in heme degradation [7]. This knowledge is important for the production of potential drugs that by activation of HO-1 expression can relieve various inflammatory diseases. PPARγ, which belongs to the PPAR family of ligand-inducible transcription factors, was very well reported to play an important role in adipogenesis and low-grade inflammation. PPARγ is active in the modulation of immunological activities and plays a significant role in facilitating immune cell differentiation and activation, as well as in changing the patterns of cytokine production and cell fates, thus reshaping the immune balance [8]. In fact, in atherosclerosis, PPARγ has been recognised as a crucial anti-inflammatory regulator primarily through the regulation of macrophage differentiation and functional polarization [9]. PPARγ Activation will bias macrophages towards the anti-inflammatory M2 phenotype resulting in inhibition of inflammation. Due to the important role of PPARγ in macrophage polarisation and anti-inflammation, PPARγ ligands could be used to combat metabolism-related inflammation and have demonstrated substantial anti-inflammatory therapeutic efficacy. Therefore, in the present study, these two proteins were selected as a potential drug target for the identification of anti-inflammatory activity of certain selected compounds by molecular docking analysis. Therefore, it is of interest to document the molecular docking analysis based binding data of furfural and isoginkgetin with heme oxygenase I and PPARγ in the context of inflammation for further consideration.

## Methodology

### Protein Preparation:

The PDB structures for Heme-oxygenase-1 (1N3U) and PPARγ (2PRG) were downloaded from the Brookhaven Protein Data Bank (www.rcsb.org) [10]. Protein structures were further cleaned using UCSF Chimera to eliminate all non- receptor atoms, including water, ion and miscellaneous compounds. The resulting structures were then saved as a pdb format.

### Ligand Preparation:

Furfural and Isoginkgetin structures have been retrieved from the PubChem database in.sdf format. And then it was translated to a.pdb format by using Online Smiles Converter. Each structure has been followed by an MMFF94 energy minimization. These collected conformations have been used as initial conformations for the docking study.

### Molecular docking:

Docking experiments have been carried out using the above-mentioned prepared target macromolecules and Furfural and Isoginggetin using the Autodock Vina program [11,12]. Docking was carried out in order to achieve a population of potential configurations and orientations for the ligand at the active site. The protein was loaded into PyRx software, producing a PDBQT file with a hydrogen protein structure in all polar residues. All the bonds of the ligands have been fixed as rotatable. Both protein-fixed ligand-flexible docking calculations have been conducted using the Lamarckian Genetic Algorithm (LGA) process. The protein target-docking site was described by setting a grid box with default grid spacing, based on the position of the native ligand. The best conformation was selected with the lowest binding energy after the docking quest was finished. Complex protein-ligand conformation interactions, including hydrogen bonds and bond lengths, were analysed using Pymol tools.

## Results and Discussion:

**Figure 1:**
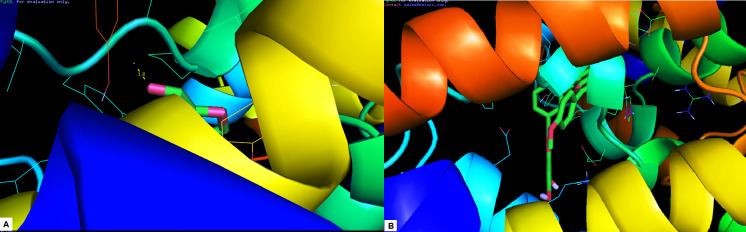
Molecular docking interaction of Heme Oxygenase I with (a) Furfural (b) Isoginkgetin.

Heme oxygenase I and PPARγ have been considered as possible drug targets and their 3D architectures have been recovered from the Protein Databank. Their binding sites have been identified. The Docking software, Autodock Vina, was used to determine the binding surface of the receptors and of the selected compounds Furfural and Isoginkgetin. Information of the docking interactions between the binding site amino acids Heme Oxygenase I and PPARγ of the two compounds were shown in Table 1. It was found to be the better docking ligand compared to Furfural with Heme Oxygenase I (Figure 1). Isoginggetin demonstrated strong binding energy as -7.9 kcal/mol relative to furfural (-6.8 kcal/mol).

**Figure 2:**
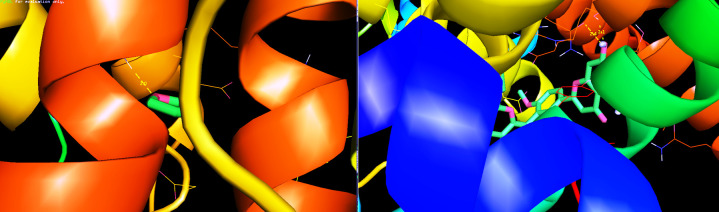
Molecular docking interaction of PPARγ with (a) Furfural (b) Isoginkgetin.

Heme Oxygenase I and PPARγ have been considered as possible drug targets and their 3D structures have been obtained from the Protein Databank. The Docking software, Autodock Vina, was used to determine the binding surface of the receptors and of the selected compounds Furfural and Isoginkgetin. The docking interactions between the binding site amino acids Heme Oxygenase I and PPARγ as shown in Table 1. Isoginkgetin was found to be the better docking ligand compared to Furfural with Heme Oxygenase I (Figure 1). Isoginggetin demonstrated strong binding energy as-7.9 kcal/mol relative to furfural (-6.8 kcal/mol). Isoginkgetin does not form any hydrogen bond interaction with Heme Oxygenase I, but the compound furfural forms a hydrogen bonding with Heme Oxygenase I between the residues of PHE-79 amino acid and N of furfural. The molecular docking analyses of PPARγ with Furfural and Isoginkgetin were seen in Table 1 and Figure 2. Among the two compounds, Isoginkgetin demonstrated very strong bonding with PPARγ in terms of the lowest bonding energy and hydrogen bonding interaction. The binding energy of Isoginggetin is-8.2 kcal/mol and the furfural has been found to be-6.4 kcal/mol with PPARγ In the hydrogen bond interaction, Isoginggetin formed two hydrogen bond interactions by GLN-444 and THE-447 amino acid residues at a distance of 2.4 Å & 2.1 Å respectively. Furfural formed a single hydrogen bond interaction with the amino acids GLN-410 at a distance of 2.0 Å. The distance between the H-bonds was less than three, suggesting favourable interactions between the ligand and the receptor. Here, however all hydrogen bond distances were below 3 such that these two compounds had favourable good interactions with the target protein PPARγ. The results propose that during the design of novel anti-inflammatory compounds, conserved amino acids should be considered to strengthen the action of the compounds against Heme Oxygenase I and PPARΔY. Induction of these targets, either natural or synthetic compounds, can represent an effective technique for reacting to liver carcinogenesis and other anti-inflammatory disorders.

## Conclusion

We document the molecular docking analysis based binding data of furfural and isoginkgetin with heme oxygenase I and PPARγ in the context of inflammation for further consideration.

## Figures and Tables

**Table 1:** Results of molecular docking studies

S. No	Compound Name	Binding Energy kcal/mol	Hydrogen bond details	Distance	No of non bonded Contacts
1	Furfural	-6.8	PHE-79 (N-O)	1.8	39
2	Isoginkgetin	-7.9	-	-	58
PPar Gamma					
1	Furfural	-6.4	GLN-410	2	
2	Isoginkgetin	-8.2	GLN-444	2.4	
			THE-447	2.1
